# Defining rhythmic locomotor burst patterns using a continuous wavelet transform

**DOI:** 10.1111/j.1749-6632.2010.05437.x

**Published:** 2010-06-10

**Authors:** Benjamin W Gallarda, Tatyana O Sharpee, Samuel L Pfaff, William A Alaynick

**Affiliations:** 1 Howard Hughes Medical Institute and Gene Expression LaboratoryLa Jolla, California; 2 Computational Neurobiology LaboratoryLa Jolla, California; 3 The Salk Institute for Biological StudiesLa Jolla, California

**Keywords:** spinal cord, locomotion, central pattern generator, continuous wavelet transform

## Abstract

We review an objective and automated method for analyzing locomotor electrophysiology data with improved speed and accuracy. Manipulating central pattern generator (CPG) organization via mouse genetics has been a critical advance in the study of this circuit. Better quantitative measures of the locomotor data will further enhance our understanding of CPG development and function. Current analysis methods aim to measure locomotor cycle period, rhythmicity, and left–right and flexor–extensor phase; however, these methods have not been optimized to detect or quantify subtle changes in locomotor output. Because multiple experiments suggest that development of the CPG is robust and that the circuit is able to achieve organized behavior by several means, we sought to find a more objective and sensitive method for quantifying locomotor output. Recently, a continuous wavelet transform (CWT) has been applied to spinal cord ventral root recordings with promising results. The CWT provides greater resolution of cycle period, phase, and rhythmicity, and is proving to be a superior technique in assessing subtle changes in locomotion due to genetic perturbations of the underlying circuitry.

## Introduction

**Figure 1:**
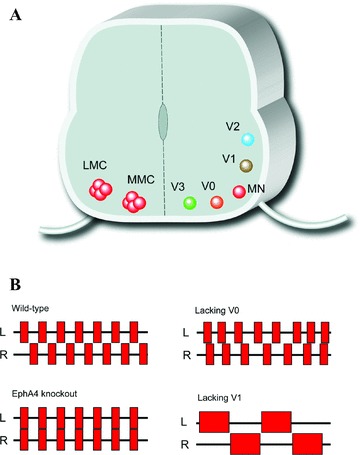
Classes of spinal neurons and locomotor output. (**A**) Diagram of a cross section of the lumbar spinal cord with medial and lateral motor pool divisions (MMC and LMC) and motor neurons; contralaterally projecting V0 interneurons, ipsilaterally projecting V1 interneurons; ipsilaterally projecting V2 interneurons, and contralaterally projecting V3 interneurons in their approximate locations in the ventral half of the spinal cord. (**B**) Diagrams of locomotor output in: wild-type showing normal left–right alternation; EphA4 knockout showing left-right synchrony; V0-deleted (*Dbx1* null) showing a perturbation of left-right coordination; and V1-deleted (*En1-DTA* or *Pax6* null) showing an increase in locomotor cycle period.

In the study of locomotion, statistical and genetic techniques have been advanced in the past two decades to improve the understanding of the circuits underlying this ubiquitous behavior. Kjaerulff and Kiehn applied circular statistics to measure the strength of right and left motor burst phase relationships and by precisely lesioning the spinal cord, they located the rhythmic centers producing the coordinated locomotor output.^1^ The application of mouse genetics to manipulate classes of spinal neurons involved in establishing locomotor circuitry has been similarly useful (Fig. 1). For example, the elimination of an axon-guidance factor, *EphA4,* results in left and right sides of the spinal cord operating synchronously to produce a hopping phenotype.^2^ Targeting transcription factors that label broad, developmentally-defined interneuron populations has illuminated the role of interneuron classes. Specifically, Evx1-positive V0 interneurons are involved in contralateral coordination, En1-positive V1 interneurons regulate the speed of locomotor output, and loss of Chx10-positive V2a interneurons causes greater variability in cycle period and burst amplitude as well as a loss of left–right coordination.^3^–^6^

**Figure 2:**
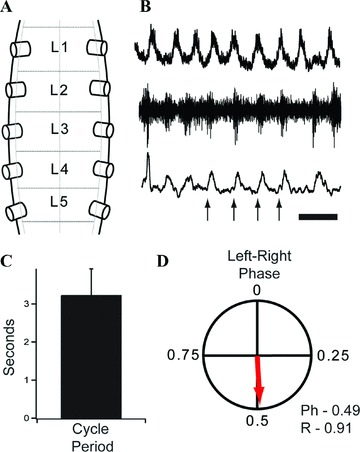
Conventional data analysis. (**A**) Diagram of the ventral surface of the lumbar spinal cord in which entire ventral roots are drawn into suction electrodes and fictive locomotion is elicited with 10 μM NMDA and 20 μM serotonin. (**B**) Conventional preprocessing of ventral root recording data for analysis. *Upper trace*: the unfiltered neurogram recorded from DC to 1 kHz, amplified 1000×, and digitized at 10 kHz. *Middle trace:* filtered with a 40 Hz high-pass filter. *Lower trace*: 40 Hz high-pass filter, then rectified and smoothed with a 5 Hz low-pass filter. The onset of randomly selected bursts (*arrows*) are then used to calculate cycle period (average of burst onset to next burst onset) and phase (average of burst onset of reference trace to average burst onset of comparison trace). (**C**) Average cycle period, mean ± SEM. (**D**) Average phase where the vector direction (Ph) indicates phase, 0.0 indicates synchrony, 0.5 indicates alternation, and vector length (R) indicates strength of phase. Scale bar equals 5 s.

Despite these advances in manipulating and recording locomotor activity, relatively little has been done to improve analysis of locomotor recordings since the seminal studies by Kjaerulff and Kiehn.^1^ The most common method of extracting useful information from ventral root recordings of neonatal spinal cords during fictive locomotion involves band-pass filtering, rectifying, and smoothing or integrating the data. This is followed by identification of burst onsets and based on a small number of randomly selected bursts (∼25), cycle period and phase information are calculated (Fig. 2).

**Figure 3:**
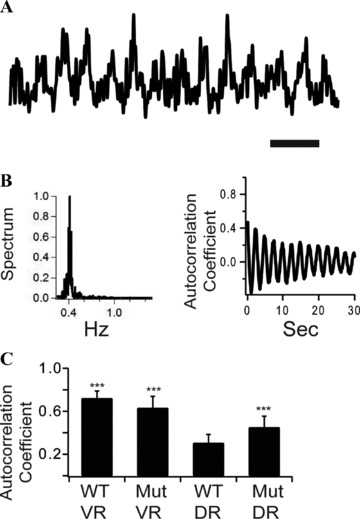
Other data analysis methods. (**A**) Raw data recorded and processed as in Figure 2 used in alternative analysis methods in *Panel **B***; bar equals 5 s. (**B**) Power spectral analysis is a measure of the dominant frequencies in the data but does not provide timing information; power spectrum of data in A normalized to 1; autocorrelation is a measure of rhythmicity in the data generated by comparing the raw data to itself; autocorrelation plot normalized to 1 (at time equals 0 s, not shown). (**C**) Autocorrelation coefficient statistical analysis on ventral and dorsal root recordings in wild type and mutant mice. Mutant dorsal root recordings show significant increase in rhythmic output due to ectopic innervation of dorsal root ganglion with motor nerves; ***indicates significantly greater than random, ANOVA followed by Dunnett's Test, *P* < 0.001. VR = ventral root; DR = dorsal root (Panel **C** adapted from Reference 8, used with permission).

Although these methods have proven well suited to detect gross changes in locomotor output, they are not well suited for analysis of trends in locomotion over longer time periods or the detection of subtle departures from control conditions. In light of this, several attempts have been made to introduce new analytical methods. For example, auto- and cross-correlation have been used as measures of rhythmicity^7^,^8^ and power spectral analysis has been applied to determine frequencies of bursting (Fig. 3).^9^ These analyses, however, are sensitive to changes in data set processing and selection, and none have significantly improved upon the traditional methods. To improve the analysis of CPG activity it is desirable to employ analytical tools that are: (1) easily automated; (2) objective and non-arbitrary; and (3) quantitative and sensitive, especially when applied to larger data sets, that is, longer recording sessions.

## Results and discussion

### Identification of rhythmic patterns using a continuous wavelet transform

**Figure 4:**
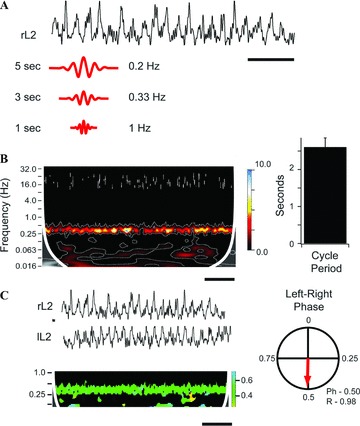
Continuous wavelet transform. (**A**) *Upper trace:* right lumbar level 2 recording recorded from DC to 1 kHz, high-pass filtered (40 Hz), rectified, and low pass filtered (5 Hz). *Lower traces:* examples of Morlet wavelet bases of three different scales. Data are convolved with similar wavelets of 100 different scales to produce the 2D plot in *Panel **B***. Scale bar equals 10 s. (**B**) Wavelet transform of data in *Panel **A*** derived with 100 scales from 0.03 to 64 s or 0.016–32 Hz with cone of influence (*solid white line*, see text for details) denoting area not analyzed due to edge effects. Significance contour (*dotted white line*) indicates areas of significant power above white noise simulation (power is indicated by color-scale gradient that is normalized to standard deviations above white noise). The *y*-axis is presented in a logarithmic scale (log2). Average cycle period is calculated from region within significance contours of wavelet transform. Scale bar equals 50 s. (**C**) Left and right lumbar level 2 data and resulting phase of cross wavelet transform superimposed on significant regions of cross wavelet transform with cone of influence (*solid white line*; only scales from 0.125 to 1 Hz shown), color-scale gradient indicates direction of vector in phase plot from 0 to 1 (only 0.4–0.6 shown); average phase circular plot derived from phase of cross wavelet transform within significant regions (as in Fig. 2C). Scale bars equal 10 s (raw data) and 50 s (cross-wavelet transform).

A recent advance that meets the criteria needed for accurate quantification of CPG activity is the use of the continuous wavelet transform (CWT).^10^ The CWT is a method of time-frequency analysis, localizing the dominant cycle periods or frequencies of the signal in time. It works by comparing the input signal across its length in time (for example, a ventral root recording—Fig. 4) to a wavelet basis—a function with zero mean, localized in time and frequency space—and plotting the resulting convolution.^11^ The wavelet basis is then scaled and iteratively compared to the input signal (for example, 100 scales from 0.016 to 32 Hz—Figs. 4A and B). The resulting plot displays regions where the wavelet basis is best matched to the signal, with frequency as the *y*-axis (typically plotted in a logarithmic scale), time as the *x*-axis, and power as the color map (Fig. 4B). When using the Morlet wavelet, a complex function, phase and frequency information are determined simultaneously. By comparing the phase of one trace with that of another, a continuous phase relationship can be determined over time (Fig. 4C). Overall cycle period and phase can then be derived (as in Figs. 2C and D). One additional benefit to the CWT is that regions of significant power can be isolated relative to an appropriate noise simulation of the input data (for details, see References 10 and 11). These are plotted as dotted white contours in Figure 4B.

The time-frequency analysis accomplished by the CWT has several advantages over previous methods. First, the CWT can be entirely automated. Much of the previously reported analysis is done manually, or is only partially automated. Because the CWT derives its results from an entire data set, there is no need for user-intervention to select parts of the data set amenable to analysis. Furthermore, although the CWT is sensitive to abrupt fluctuations in the unprocessed data, the standard preprocessing of data to remove slow fluctuations and flatten the baseline has little effect on the results. This negates the need to preprocess data, improving automation and speed of analysis. Second, the CWT, being a continuous measure, is able to assess changes in locomotor output over time due to treatments or perturbations of the system. Whether traditional lesion studies or pharmacological treatments, or newer techniques such as allatostatin signaling or channelrhodopsin/light-based perturbation of neuronal activity,^5^,^12^ the CWT can accommodate a dynamic measure of changes in output. This is superior to a simple summary of pre- and posttreatment metrics. Third, the CWT is scalable in time, so that it extracts cycle period and phase information with appropriate temporal resolution. For example, one may be concerned with a change in cycle period of motor output from 1.0 to 1.2 s, but is less likely to be as concerned with a change from 20.0 to 20.2 s. The CWT addresses this, especially when using scales on power-of-2 intervals, by allowing the user to select only the desired scales with increasing intervals between them. This is superior to other time-frequency analyses such as a short-term Fourier transform, which uses a fixed interval based on the selected window size (see Reference 10, Fig. 2). In their paper, Mor and Lev-Tov point out that the CWT does not depend on burst detection to quantify cycle period or phase. However, should one desire to identify bursts for further analysis, the CWT is an excellent tool for burst detection as well. Instead of computing the complex transform, a real wavelet transform highlights peaks and valleys where the wavelet basis is in-phase and out-of-phase with the raw signal. By following the trend of the cycle period relevant to bursting, individual bursts can be identified and extracted for further analysis.

### Statistical considerations

There are several ways to quantify the strength of the peaks in the wavelet transform. Monte-Carlo simulations, consisting of thousands of generated samples of noise with similar power spectra to the raw data (white noise being most appropriate for filtered and smoothed spinal cord ventral root recordings), can be averaged to produce a wavelet transform of random noise.^10^ The wavelet transform of the raw data can then be compared to this and any peaks above the noise are considered significant. There are also equation-based approximations of noise spectra that can be used with similar results as Monte Carlo simulations that drastically reduce computation time.^11^ Others have pointed out possible shortcomings in the noise-based detection of significant regions of the wavelet transform and have suggested other mathematical models for identifying these regions.^13^,^14^ By selecting the appropriate method of assessing the strength of signal in the wavelet transform, quantification of regions of significant power can be automated. In sum, the CWT is an advance from previous methods as it demonstrates a more objective, sensitive, and quantitative analysis of electrophysiological signals that can be easily automated.

### Defining flexor–extensor phase using CWT

**Figure 5:**
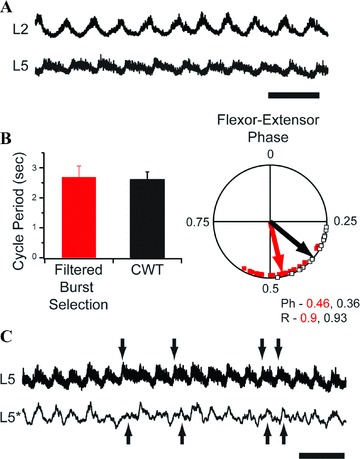
Continuous wavelet transform results. (**A**) Ipsilateral L2 and L5 recordings from DC to 1 kHz, not filtered. (**B**) Comparison of cycle period and phase calculated by conventional methods (*red squares* in phase plot offset slightly for clarity) or with CWT (*open squares*) using 800 s of data, a portion of which is shown in *Panel **A***. Cycle period is mean + SEM; phase plot includes mean vector and 25 individual data points representing phase values at 25 bursts in the raw data. (**C**) *Upper trace*: DC to 1 kHz, not filtered. *Lower trace*: filtered, rectified, smoothed L5 data. *Arrows* indicate shifts due to processing that account for the change in phase between conventional methods and CWT in *Panel **B***. Scale bars equal 5 s.

What then are the results of applying the CWT to locomotor data from typical lumbar level recordings? The average cycle period and phase are indistinguishable from those obtained through conventional methods, in most instances (Figs. 4 and 5), and the CWT is achieving these values when applied to data sets 20 or more times as long as those used conventionally. Such an increase in the amount of data analyzed will improve statistical analysis of the results. Although most of the results of CWT-based analysis accurately reproduce those previously reported, an interesting difference appears when assessing flexor–extensor phase in late-embryonic/early-postnatal mice. Flexor–extensor phase has been generally defined by previous studies using previous methods as 0.5—exact alternation, similar to left–right phase (Figs. 2D and 5B). This value is calculated from data recorded at lumbar level 2 (L2) and lumbar level 5 (L5) ventral roots, which are thought to innervate predominantly flexor and extensor muscles, respectively. The CWT, however, reports a flexor–extensor phase value of 0.36, a significantly different result (Fig. 5B). This is not an artifact of the CWT, as left–right phase relationships calculated by the CWT are 0.5 (Fig. 2D). Rather, this discrepancy between the CWT and conventional methods is due to filtering, rectifying, and smoothing the raw data for conventional analysis (Fig. 2B). The result of preprocessing raw data in such a way is an effective integration of high-frequency motor bursting; whereas low-frequency phenomena such as the population of motor neuron membrane depolarizations, recorded in DC, are discarded.^15^ Thus, when the onset of motor bursting is not aligned with the onset of DC depolarization, a phase shift can occur (Fig. 5C). Furthermore, conventional methods define a specific point, typically burst onset, in order to assess phase. In contrast, the CWT defines phase throughout the burst cycle, giving a more comprehensive measure of this relationship between the two signals. The cumulative effect of these two points is that the actual phase of L5 is obscured by preprocessing the data and using a single point for each burst to define phase. This effect is likely more significant in L5 data. A similar effect is not noticed in L2 because the onset of motor bursting aligns with the onset of DC depolarization in L2 output; and this shift in L5 with corresponding lack of shift in L2 explains the change in L2–L5 phase. Interestingly, the 0.36 flexor–extensor phase value detected at E18.5-P0 shifts to 0.5 at P2 (unpublished observation BWG, WAA). This suggests that the CWT is capable of detecting postnatal changes in flexor–extensor locomotor output. The basis for this postnatal phase shift remains to be defined.

## Conclusions

In summary, the CWT improves upon previous methods in the following ways. First, the CWT is easily automated, objective, and quantitative. For example, CWT produces quantitative measures of rhythmicity by virtue of detection of frequencies compared to noise-simulation cutoffs for statistical significance. This is a substantial improvement over autocorrelation that is dependent on both preprocessing and the length of data selected for analysis (Fig. 3B). Second, unlike previous methods that only provide a summary of important measures such as cycle period and phase, the CWT extracts this information continuously across the length of a recording, which can be averaged to produce conventional summary statistics or examined to see if measures change over time. Unlike power spectral analysis, which is a measure of frequency only, and can change drastically based on differences in data processing and selection (Fig. 3B), the CWT measures frequencies or cycle periods in the data continuously. Third, the CWT is a more precise measure of electrophysiological data, uncovering a previously unappreciated flexor–extensor phase relationship obfuscated by conventional methods (Fig. 5). Some practical considerations related to application of the CWT follow. Instead of selecting subsets of bursts, only clearly aberrant artifacts need to be removed from the raw data. There are edge effects that need to be considered at the beginning and end of a CWT of raw data that are proportional to the size of the scale being applied. These are known as the cone of influence (COI),^11^ but this region can be easily plotted with the CWT and these areas can be discounted from analysis (Fig. 4B). Finally, while electrophysiological recordings are typically processed according to the steps described in Figure 2, we have found that this preprocessing does not greatly improve the results of a CWT, and unprocessed data can be used for analysis. This matter of preprocessing data clearly alters the phase relationship when comparing the output from different lumbar levels (see Fig. 5).

Prospectively, use of the CWT will allow for additional analyses to be considered. For example, the CWT can be used to rapidly define hundreds of individual motor bursts in long data sets. Such large numbers of motor bursts could be extracted and subjected to other statistical analyses, such as principal component analysis (PCA).^16^ As motor bursts are generally considered in an almost binary fashion—“on” or “off”—this added benefit of using the CWT may uncover new dynamics in locomotor output not previously appreciated. And because the data analyzed through the CWT need not be preprocessed, subsequent manipulations of the data are still available.

The study of locomotion will benefit tremendously from advances in data analysis. New standards for characterizing abnormal locomotor output that extend beyond summary statistics will be critical in defining the changes in circuitry produced by subtle genetic manipulations of the spinal cord. These findings may then serve to better inform computer models and interpretation of experimental results. The CWT appears to meet the necessary criteria for emerging analysis methods of electrophysiological data that offer a more comprehensive and informative view of locomotor activity.
